# *CDH1* Missense Variant *c*.*1679C>G* (p.T560R) Completely Disrupts Normal Splicing through Creation of a Novel 5’ Splice Site

**DOI:** 10.1371/journal.pone.0165654

**Published:** 2016-11-23

**Authors:** Zarina Yelskaya, Ruben Bacares, Erin Salo-Mullen, Joshua Somar, Deborah A. Lehrich, Grace-Ann Fasaye, Daniel G. Coit, Laura H. Tang, Zsofia K. Stadler, Liying Zhang

**Affiliations:** 1 Department of Pathology, Memorial Sloan Kettering Cancer Center, New York, New York, United States of America; 2 Department of Medicine, Memorial Sloan Kettering Cancer Center, New York, New York, United States of America; 3 Department of Hematology Oncology, Walter Reed National Military Medical Center, Bethesda, Maryland, United States of America; 4 Department of Surgical Oncology, Walter Reed National Military Medical Center, Bethesda, Maryland, United States of America; 5 Department of Surgery, Memorial Sloan Kettering Cancer Center, New York, New York, United States of America; Odense University Hospital, DENMARK

## Abstract

Disease-causing germline mutations in *CDH1* cause Hereditary Diffuse Gastric Cancer (HDGC). For patients who meet the HDGC screening criteria, the identification and classification of the sequence variants found in *CDH1* are critical for risk management of patients. In this report, we describe a germline *CDH1 c*.*1679C>G* (p.T560R) variant identified in a 50 year old man who was diagnosed with gastric cancer with a strong family history of gastric cancer (one living brother was diagnosed with gastric cancer at 63 and another brother died of gastric cancer at 45). cDNA analysis, involving fragment analysis and cloning, indicated that the p.T560R mutation created a novel 5’ splice donor site, which led to a novel transcript with a 32 nucleotide deletion in exon 11. This abnormal transcript putatively produces a truncated *CDH1* protein (E-cadherin) of 575 amino acids instead of 882. We also demonstrated that the variant completely abolishes normal splicing as the mutant allele does not generate any normal transcript. Furthermore, the *CDH1 c*.*1679C>G* (p.T560R) variant segregated with gastric cancer in all three family members affected with gastric cancer in this family. These results support the conclusion that *CDH1 c*.*1679C>G* (p.T560R) variant is a pathogenic mutation and contributes to HDGC through disruption of normal splicing.

## Introduction

*CDH1* gene encodes for E-cadherin transmembrane glycoprotein expressed on epithelial tissue and is responsible for calcium-dependent cell-to-cell adhesion [1]. E-cadherin protein forms intercellular adhesion structures that act as tumor suppressor preventing tumor invasion and metastasis. Germline mutations in *CDH1* cause an autosomal dominant, inherited gastric cancer susceptibility syndrome, known as Hereditary diffuse gastric cancer (HDGC, OMIM #137215) [2, 3]. In *CDH1* mutation carriers, the cumulative risk of gastric carcinoma by 80 years of age is 70% in men and 56% in women, and the risk of breast cancer for females was 42% [4]. Genetic testing for *CDH1* germline mutations is critical for patients with early onset gastric cancer and/or a strong family history because it affects management of this disease. For patients who carry a clinically significant mutation in *CDH1*, prophylactic total gastrectomy remains the only treatment option as a preventive measure to reduce the risk of developing gastric cancer [5, 6].

Since not all variants are disease causing, they need to be routinely assessed for pathogenicity once genetic testing results are obtained. *CDH1* truncating mutations, such as nonsense mutation and small insertions/deletions, and alterations of a canonical dinucleotide splice donor/acceptor sequences that affect the GU-AG rules, are most often straightforward to interpret because these mutations are usually pathogenic. However, assessment of non-truncating sequence variants in tumor suppressor genes can be challenging when these changes are subtle and are unknown to alter function sufficiently to predispose to cancer development. Although the focus is usually placed on its effects on protein structure and function, single nucleotide substitutions within exons can also have significant impact on mRNA processing, and disrupt protein function [7, 8]. Substitution mutations and synonymous alterations should always be studied for their potentials to disrupt pre-mRNA splicing. They may affect the canonical splice sites or splicing enhancers (ESEs), create novel splicing sites, activate cryptic splicing sites, and ultimately have a detrimental effect on protein function [9].

In this report, we describe a gastric cancer family with a rare missense *CDH1* substitution variant, *c*.*1679C>G* (p.T560R). cDNA functional analysis indicated that the p.T560R variant completely abolishes normal splicing by creating a novel 5’ splice donor site, which led to a novel transcript with a 32 bp deletion in exon 11 and premature protein truncation. Furthermore, we have demonstrated that the mutation co-segregates with gastric cancer in three affected family members, which supports the pathogenicity of this variant. This variant has been reported once in a young patient affected with gastric cancer with no family history of cancer. However, no further functional studies were performed [10].

## Subjects and Methods

### Subjects

**Fig 1:**
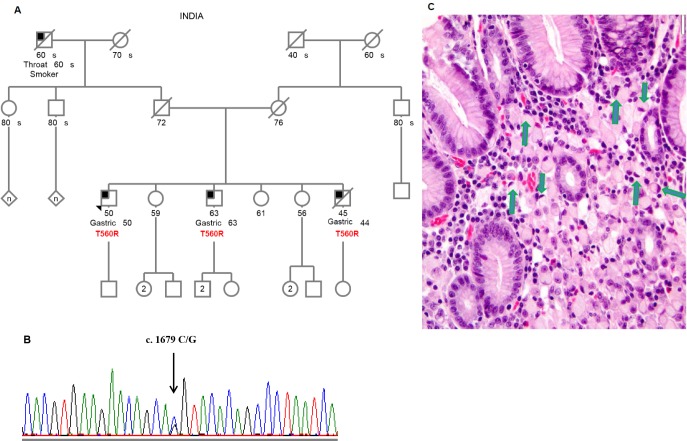
Patient pedigree, *CDH1* mutation and H&E image of diffuse gastric cancer. (A) The patient (indicated with the arrow head) is a 50 year old man who was diagnosed with gastric cancer at the age of 50. In his generation, three members were diagnosed with gastric cancers (one brother died of gastric cancer at 45, another brother was diagnosed with gastric cancer at 63). All three brothers affected with gastric cancer were determined to have the *CDH1 c*.*1679 C>G* p.T560R variant. (B) our patient’s germline *CDH1* sequencing demonstrating the *CDH1 c*.*1679C>G* variant. (C) Haematoxylin and eosin stain (H&E stain) of patient’s biopsy specimen showing infiltrating adenocarcinoma poorly differentiated with mucinous and signet ring cell features. The signet ring cells are characteristic of hereditary diffuse gastric cancer (HDGC). They contain large amount of mucin which pushes the nucleus to the cell periphery (see arrows).

We report on a 50 year old man of Indian descent who was diagnosed with gastric cancer at age of 50. In his generation, three members including the proband were diagnosed with gastric cancer (one brother died of gastric cancer at 45, another brother was diagnosed with gastric cancer at 63) (Fig 1A). The *CDH1 c*.*1679C>G* (p.T560R) was identified in the patient through *CDH1* full gene sequencing analysis at the Diagnostic Molecular Genetics Laboratory at Memorial Sloan-Kettering Cancer Center (MSKCC). The brother affected with gastric cancer at 63 was identified to carry the same *CDH1* variant through testing in a reference lab, which was initially classified as a variant of uncertain significance. Patient’s father had a reported history of gastric ulcers.

Given the uncertain clinical significance of this variant, the patient and the living brother affected with gastric cancer elected to participate in a research study to further characterize the variant. The research protocol was reviewed and approved by Memorial Sloan Kettering Cancer Center institutional review board. The participants provided their written informed consent to participate in this study. The institutional review board approved the consent procedure. A peripheral blood sample from the patient and his brother was collected using the PAXgene Blood RNA tube and submitted for analysis. Control RNAs were extracted from eight unrelated individuals seen at Memorial Sloan Kettering Cancer Center who do not carry the *CDH1 c*.*1679C>G* (p.T560R) variant.

### cDNA Synthesis

Total RNA was extracted from whole blood using the PAXgene Blood RNA Kit (PreAnalytiX, Qiagen, Valencia, CA) and was subsequently used for cDNA synthesis (SuperScript III First-Strand Synthesis System, Invitrogen Life Technologies, Carlsbad, CA). *CDH1* exons 10–12 were amplified, the sequence of the forward primer is 5’-TCACATCCTACACTGCCCAG-3’ and the sequence of the reverse primer is 5’-TTCGAGGTTCTGGTATGGG-3’. Each PCR reaction consisted of 35μl SIGMA REDTaqReady Mix 1x, 2μl of 10μM forward and reverse primers, 2μl of cDNA and water to a final volume of 50μl. Cycling conditions were 96°C for 5min, 94°C for 30 sec (40x), 58°C for 45 sec (40x), and 72°C for 60 sec (40x), with a final extension at 72°C for 5 min.

### Fragment analysis

cDNA products were subject to the same primer sequences and PCR conditions as mentioned above, with the exception of forward primer sequence used for this reaction was labeled with 5’ 56-JOEN fluorophore. The RT-PCR products amplified using JOE fluorophore labeled primer were subjected to fragment analysis on 3730 Genetic Analyzer (Applied Biosystems, Foster City, CA) with the internal lane standard 600 (ILS 600) (Promega Corporation, Madison, WI), used as a DNA ladder to assign correct sizes to DNA fragments. The percentages of the different transcripts of the total transcripts were calculated based on the peak heights of individual fragments.

### Cloning

To determine if the mutant allele created alternative transcripts, RT-PCR products were cloned into pCR4 TOPO vectors (Invitrogen, Carlsbad, CA), following manufacturer procedures (Invitrogen, Carlsbad, CA). DNA from colonies was amplified using the *CDH1* primers covering cDNA regions of exons 10–12 and subjected to direct DNA sequencing analysis using forward and reverse primers (BigDye Terminator v3.1 Cycle Sequencing kit and 3730 Genetic analyzer, Applied Biosystems, Foster City, CA).

### Statistical analysis

Two-tailed unpaired Student's *t*-test was used to perform statistical analysis. In all analyses, *p*<0.02 was required for statistical significance.

## Results

### Patient and family history

Our proband is a 50 year old man of Indian descent who was diagnosed with gastric cancer at age of 50. In his generation, three members including the proband were diagnosed with gastric cancer (one brother died of gastric cancer at 45, another brother was diagnosed with gastric cancer at 63) (Fig 1A). The proband pursued clinical genetic testing and we identified a *CDH1 c*.*1679C>G* (p.T560R) variant through *CDH1* full gene sequencing analysis in our laboratory (Fig 1B). Patient’s stomach biopsy specimen revealed infiltrating adenocarcinoma poorly differentiated with mucinous and signet ring cell features (Fig 1C). Both living and deceased brothers had poorly differentiated gastric adenocarcinoma.

### Variant *In silico* Analysis

**Fig 2:**
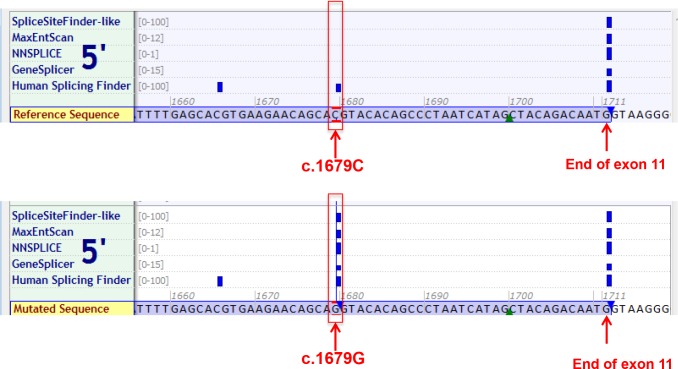
*In silico* prediction of the *CDH1* p.T560R variant. Prediction of the potential effects of the *CDH1 c*.*1679 C>G* (p.T560R) alteration on normal mRNA splicing. Top panel: reference sequence; bottom panel: mutated sequence. The text at the left of each panel represents the names of the tools used to predict splice site strength. The number ranges indicate the strength of each target sequence in each prediction tool with the upper range being the perfect match. The reference sequence and mutated sequences are shown at the bottom of each panel with nucleotide positions indicated above. Blue vertical bars indicate 5’ (donor) site. The height of each bar is proportional to the maximum possible score computed by the corresponding algorithm.

Sequence data spanning the CDH1 locus for Homo sapiens [Chromosome 16: 68,737,225–68,835,548] was obtained from the Ensembl Genome Browser (http://www.ensembl.org/index.html). CDH1 cDNA and protein sequences of selected species were selected for multiple sequence alignment using Alamut. For these comparisons, Homo sapiens was considered the base sequence. Multi-species comparative genomic analysis was used to identify sequence homology at the *CDH1 c*.*1679 C>G* (p.T560R) variant site in ten distantly related species: Human, Chimpanzee, Northern white-cheeked gibbon, Olive Baboon, Rat, Mouse, Dog, Platypus, Chicken and Frog. This analysis indicated the *CDH1* p.560T is well-conserved across these species (data not shown), with p.560T present in 10 out of 10 species analyzed, making the change to p.560R possibly detrimental to CDH1 gene function. *In silico* analysis using Polyphen, SIFT, AlignGVGD and Mutation Taster predicted *CDH1 c*.*1679 C>G* (p.T560R) to be “probably damaging”, “damaging”, “most likely to interfere with function” and “disease causing”, respectively. In addition to the predictions on the E-cadherin function as a substitution variant, we also evaluated its potential effects on splicing. We used Alamut software, which incorporates five tools to predict the potential effects of *CDH1 c*.*1679C>G* on normal mRNA splicing. All five tools, with high scores in 4 out of 5, predicted that the missense variant created a novel splice site that is consistent with the CAG/GU consensus 5’ splice site (Fig 2).

### Fragment Analysis of RNA transcripts

**Fig 3:**
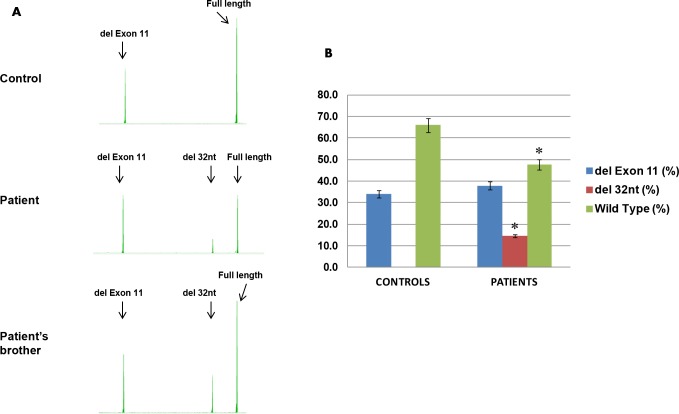
Semi-quantitative fragment analysis of *CDH1* RNA transcripts. (A) RT-PCR fragments generated from *CDH1* spanning exons 10–12 cDNAs from patient, patient’s brother, and eight controls (results from one representative fragment analysis run were shown) were analyzed by capillary electrophoresis (3730 Genetic Analyzer). The three peaks observed (from left to right) are: del Exon 11, del 32nt, and Full length. (B) Quantification of the three transcripts in controls and affected patients (the proband and his brother). The peak heights for the three transcripts in the patient and patient’s brother were averaged because the ratios are similar. The peak heights for the eight control samples were also averaged. Blue, red and green bars represent percentages of fragments with exon 11deletion, a deletion of 32 nucleotides within exon 11, and wild type fragments. Error bars represent standard error in comparing controls and patients for each fragment. Asterisks represent statistical significance between controls and patients.

Based on the *in silico* prediction results and patient’s strong personal and family history, we decided to evaluate the effect of the *CDH1 c*.*1679 C>G* on mRNA splicing by amplifying regions of *CDH1* using cDNA derived from the patient, patient’s living affected brother (who also carries the same variant), and individuals who do not carry this variant. The PCR was designed to generate fragments containing exons 10–12 of *CDH1*, which are the exons most likely to be affected by the mutation. Fragment analysis of RT-PCR products spanning *CDH1* exons 10–12 resulted in three transcripts. One transcript of predicted size of RT-PCR product, “Wild type”, was observed in the controls as well as in the patient and his brother. It is the fragment generated by the wild type allele(s). Another transcript observed in the controls, patient and his brother, was shorter by 146 base pairs than the full-length fragment, “del Exon 11.” This fragment is consistent with alternative *CDH1* transcript that skips exon 11. Interestingly, we observed a novel transcript that is unique in the patient and his living brother: 32 nucleotides shorter than the full-length fragment, “del 32nt” (Fig 3A).

We then calculated the ratios of the alternative/novel transcripts and the exon 11 full-length transcript, comparing the amounts of these transcripts to the sum of all transcripts, taken as an approximation for the total *CDH1* transcript level. Percentages of each the transcripts obtained within each of the eight control samples and affected patients (patient and patient’s brother) were averaged and depicted in Fig 3B. The *CDH1* exon 11 full length transcript represented 48% of the total *CDH1* transcript level in the patient and his brother, which was significantly reduced than that in the controls (66%, *p*<0.01). The *CDH1* del 32 nt transcript, which is absent in controls, represented 15% of the total *CDH1* transcript level in patient and his brother. The amounts of exon 11 alternative transcript were similar in the brothers and control samples (Fig 3). Based on these results, we speculate the *c*.*1679 C>G* (p.T560R) variant activates a cryptic donor site within exon 11 and generates an out-of-frame novel transcript (*CDH1* del 32 nt, Fig 3). The deletion of 32 nucleotides at the 3’ end of exon 11 presumably leads frameshift and protein truncation.

### Cloning Analysis of RNA transcripts

**Fig 4:**
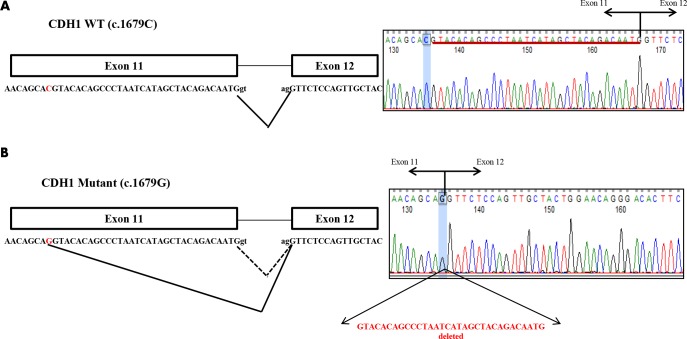
*CDH1 c*.*1679 C>G* (p.T560R) mutant allele creates a novel splice site that results in a 32 nucleotide deletion of the 3’ end of exon 11. (A) Sequence adjacent to wild type allele, *CDH1 c*.*1679 C*, indicating normal splice site. Electropherogram data of normally spliced cDNA with wild type allele highlighted in blue. (B) Sequence adjacent to mutant allele, *CDH1 c*.*1679 G*, indicating aberrant splice site produced by mutant allele (solid lines) and location of normal splice site (dashed lines). Electropherogram data of aberrantly spliced cDNA with the mutant allele highlighted in light blue.

To determine whether the *CDH1 c*.*1679 G* mutant allele completely disrupts normal splicing, i.e., whether the mutant allele is able to generate any *CDH1* exon 11 full length transcripts with the T560R missense mutation, we cloned the RT-PCR products into the TOPO sequencing vector and then sequenced 21 colonies. All 21 clones from the patient and his brother contained the normal “C” allele in the full length transcript, indicating that the mutant allele was unable to generate any normal transcript (Fig 4A). However, all clones with the 32 nt deletion contained the mutant “G” allele (Fig 4B). These results indicate that the mutant “G” allele completely abolishes normal splicing through activation of a cryptic splice site within exon 11 of *CDH1*. We also confirmed the exon 11 deletion by Sanger sequencing (data not shown).

### Segregation studies

To investigate whether this *CDH1* variant segregates with gastric cancer in the family, we obtained *CDH1* testing results of the 63 year old brother (tested in a reference lab) which indicates he carries the *CDH1 c*.*1679 C>G* (p.T560R) variant. We also obtained the deceased brother’s tumor tissue to test for this familial mutation. He also carried the same *CDH1* variant (data not shown). Taken together, the *CDH1 c*.*1679 C>G* (p.T560R) variant was detected in all three brothers affected with gastric cancer. Co-segregation of this allele with gastric cancer is consistent with the expectation for damaging alleles with high disease penetrance.

## Discussion

Recent advances in molecular diagnostic sequencing technologies have resulted in generation of extensive discoveries in DNA alteration. Identified genetic variants need to be assessed and classified to determine whether they contribute to disease. Even though bioinformatic analysis can be useful in predicting the biological consequences of missense variants, studies have revealed many challenges and inconsistencies among existing tools [11]. Predictions tools for missense variants using PolyPhen, SIFT, MutationTaster and AlignGVGD all predicted *CDH1 c*.*1679 C>G* (p.T560R) variant disrupts normal protein function. Additional *in silico* analysis predicted that this genomic alteration created a novel 5’ splice donor site that matches with the CAG/GU consensus sequence. Although the bioinformatics tools predicted *CDH1 c*.*1679C>G* (p.T560R) variant might affect the normal function of the gene, they do not tell whether it creates an amino acid substitution in the mature protein or disrupts normal splicing, or both. Our studies proved it is through the disruption of normal splicing and demonstrate the importance of splicing functional studies in the interpretation and classification of missense variants.

Exon-intron boundaries are identified by the recognition and binding of U1 snRNP to the 5’ splice site, SF1/BBP to the branch site and the U2AF65/U2AF35 complex to the 3’ splice site [12]. U1snRNP binding to the 5' splice site through base pairing between the single stranded terminal sequence of the U1 RNA molecule and the loosely conserved stretch of nucleotides at the 5' splice site, CAG/GUAAGU, initiates spliceosome assembly. The GU dinucleotide in this 9 nt consensus sequence, which can be expanded to include 11 base pairs (CAG/GUAAGUAU), is predominantly conserved in the vast majority of the 5' splice sites that make up the mammalian GT-AG introns [12, 13]. Roca et al. 2013 review reported on updated 5’ splice site motifs based on a collection of 201,541 human 5’ splice site sequences presenting variation frequencies among the four nucleotides in each of the positions of the 11nt consensus sequence and showed that although the GU dinucleotide in this 11nt consensus sequence are predominantly conserved, the remaining 9 positions are more variable [13]. In the case of *CDH1 c*.*1679 C>G*, this missense variant changed the c.1679 C nucleotide with the previously splice site unrecognizable sequence to c.1679 G nucleotide. This change created a novel spliceosomes recognizable sequence of CAG/GU (Fig 4) forming a novel 5’ splice site within exon 11, leading to the deletion of the 32 nucleotides at the 3’ end of exon 11.

Sequences that match the consensus splice site are common in introns and recognition of such sequences by the spliceosome leads to alternative splicing [9]. As a consequence of alternative splicing, the same pre-mRNA allows for the production of several protein isoforms [12]. Upon cloning and sequencing our transcripts, we found that one of the transcripts was completely lacking Exon 11 of *CDH1* gene (sequencing data not shown). We determined this transcript to be a result of exon skipping mode of alternative splicing, Ensembl ID (CDH1-005) ENST566612. Previous studies have shown that this transcript is overexpressed in breast, prostate, CLL, head and neck cancers as compared to the normal non-malignant cells. Its levels inversely correlate with E-cadherin expression [14, 15]. Since the exon 11 skipping leads to frameshift and nonsense mediated decay, the increase in the aberrant splicing is a mechanism of loss of E-cadherin. SFRS2 (SC35), a splicing factor, increases missplicing and downregulates E-cadherin expression in head and neck cancers [15]. In addition, *CDH1* promoter methylation, which is involved in silencing the E-cadherin expression in HDGC tumors [16], is also involved in *CDH1* missplicing in tumors [15]. Since we studied the *CDH1* exon 11 deletion in patient’s normal white blood cells and the previous studies were performed on B lymphocytes, which is one of the components of white blood cells, this might explain the fact that we observed much higher level of aberrant transcript [17].

Loss of Heterozygosity (LOH) is the most common molecular genetic alteration encountered in human cancers. We tested the tumor specimens from the patient, patient’s living brother and deceased brother. We did not observe LOH in any tumor (data not shown). This result is consistent with previous reports that epigenetic changes in HDGC primary tumors are more common, while LOH is more prevalent in metastases as second hit [16].

As mentioned above, the CDH1 gene encodes for E-cadherin transmembrane glycoprotein expressed on epithelial tissue and is responsible for calcium-dependent cell-to-cell adhesion [1]. E-cadherin proprotein coding regions consist of signal peptide, precursor peptide, extracellular domain, transmembrane domain, and cytoplasmic domain. E-cadherin molecules are synthesized as inactive propeptide precursors that need to be processed to become active mature proteins [18]. Post-translational processing of E-cadherin involves proprotein cleavage and removal of signal peptide and precursor peptide encoded by exon 1–3 and 5’end of exon 4. Initially synthesized E-cadherin proprotein are 882 amino acids. However, the E-cadherin proprotein with CDH1 p.T560R variant creates a novel 5’ splice donor site leading to a transcript with a 32 bp deletion in exon 11, shifting the codon frame, which results in a premature stop codon at position 576; thus, a truncated *CDH1* protein (E-cadherin) of 575 amino acids. Additionally, the instability of *CDH1* transcripts with germline truncating mutations such as those resulting in premature termination codons, are subject to nonsense-mediated decay, which has been correlated with earlier age of onset of gastric cancer [19]. In this case, NMD may exacerbate the reduction of aberrant *CDH1* transcripts due to the presence of both the novel del 32 nt transcript and the alternatively spliced isoform of exon 11 deletion. This led to a significant lower level of wild type *CDH1* transcripts in the affected patients comparing the controls. These results support the conclusion that *CDH1 c*.*1679C>G* (p.T560R) variant is a pathogenic mutation.

Individuals who carry a pathogenic *CDH1* mutation may benefit from a prophylactic gastrectomy, which at present, is the only effective clinical option for carriers of germline *CDH1* mutations. However, gastrectomies result in significant risks that may interfere with the patient’s quality of life. Therefore, it is critical to identify individuals at high-risk for developing diffuse gastric cancer from those with relatively low risk or population risk for developing such disease, allowing for surveillance of gastric cancer in high risk but asymptomatic individuals. High risk individuals are recommended periodic endoscopic surveillance from 40 years of age, or 5 years younger than the youngest diagnosis in the family. [1] Our study highlights the importance of assessing ambiguous variants for pathogenicity which allows patients with deleterious mutations to enter a regimen of surveillance and prophylactic resection, decisions that are completely different from those individuals with a variant of unknown significance (VUS) or benign polymorphisms.

## Conclusions

We report data from functional studies which supported that *CDH1 c*.*1679 C>G* (p.T560R) is a pathogenic variant. This mutation provides an example of a complex mechanism by which missense variants may be responsible for cancer predisposition through disruption of normal splicing rather than creating an amino acid substitution in the mature protein. Therefore, the potential disruption of normal mRNA splicing needs to be considered for exonic substitution, in both missense and synonymous variants of unknown significance.

## References

[1] Corso G, Marrelli D, Roviello F. E-cadherin germline missense mutations in diffuse gastric cancer. OA Cancer. 2013;1(1):4.

[2] Guilford P, Hopkins J, Harraway J, McLeod M, McLeod N, Harawira P, et al E-cadherin germline mutations in familial gastric cancer. Nature. 1998;392(6674):402–5. Epub 1998/04/16. 10.1038/32918 . 9537325

[3] Guilford P, Humar B, Blair V. Hereditary diffuse gastric cancer: translation of CDH1 germline mutations into clinical practice. Gastric cancer: official journal of the International Gastric Cancer Association and the Japanese Gastric Cancer Association. 2010;13(1):1–10. 10.1007/s10120-009-0531-x . 20373070

[4] Hansford S, Kaurah P, Li-Chang H, Woo M, Senz J, Pinheiro H, et al Hereditary Diffuse Gastric Cancer Syndrome: CDH1 Mutations and Beyond. JAMA oncology. 2015;1(1):23–32. 10.1001/jamaoncol.2014.168 . 26182300

[5] Fitzgerald RC, Hardwick R, Huntsman D, Carneiro F, Guilford P, Blair V, et al Hereditary diffuse gastric cancer: updated consensus guidelines for clinical management and directions for future research. J Med Genet. 2010;47(7):436–44. 10.1136/jmg.2009.074237 20591882 PMC2991043

[6] Huntsman DG, Carneiro F, Lewis FR, MacLeod PM, Hayashi A, Monaghan KG, et al Early gastric cancer in young, asymptomatic carriers of germ-line E-cadherin mutations. The New England journal of medicine. 2001;344(25):1904–9. 10.1056/NEJM200106213442504 . 11419427

[7] Morrison A, Chekaluk Y, Bacares R, Ladanyi M, Zhang L. BAP1 missense mutation c.2054 A>T (p.E685V) completely disrupts normal splicing through creation of a novel 5' splice site in a human mesothelioma cell line. PloS one. 2015;10(4):e0119224 10.1371/journal.pone.0119224 25830670 PMC4382119

[8] Zhang L, Xiao A, Ruggeri J, Bacares R, Somar J, Melo S, et al The germline CDH1 c.48 G>C substitution contributes to cancer predisposition through generation of a pro-invasive mutation. Mutat Res. 2014;770:106–11. 10.1016/j.mrfmmm.2014.10.001 . 25771876

[9] Cartegni L, Chew SL, Krainer AR. Listening to silence and understanding nonsense: exonic mutations that affect splicing. Nature reviews Genetics. 2002;3(4):285–98. Epub 2002/04/23. 10.1038/nrg775 . 11967553

[10] Benusiglio PR, Malka D, Rouleau E, De Pauw A, Buecher B, Nogues C, et al CDH1 germline mutations and the hereditary diffuse gastric and lobular breast cancer syndrome: a multicentre study. J Med Genet. 2013;50(7):486–9. 10.1136/jmedgenet-2012-101472 . 23709761

[11] Gnad F, Baucom A, Mukhyala K, Manning G, Zhang Z. Assessment of computational methods for predicting the effects of missense mutations in human cancers. BMC Genomics. 2013;14(Suppl 3):S7 Epub 28 May 2013. 10.1186/1471-2164-14-S3-S7 PubMed Central PMCID: PMC PMC3665581 23819521 PMC3665581

[12] Buratti E, Baralle D. Novel roles of U1 snRNP in alternative splicing regulation. RNA Biology. 2010;7:4:412–9. 10.4161/rna.7.4.12153 20523112

[13] Roca X, Krainer AR, Eperson IC. Pick one, but be quick: 5' splice site and the problems of too many choices. Genes & Development. 2013:129–44. 10.1101/gad.209759.112 23348838 PMC3566305

[14] Jordaan G, Liao W, Sharma S. E-cadherin gene re-expression in chronic lymphocytic leukemia cells by HDAC inhibitors. BMC Cancer. 2013;13:88 10.1186/1471-2407-13-88 23432814 PMC3586366

[15] Sharma S, Liao W, Zhou X, Wong DT, Lichtenstein A. Exon 11 skipping of E-cadherin RNA downregulates its expression in head and neck cancer cells. Molecular cancer therapeutics. 2011;10(9):1751–9. 10.1158/1535-7163.MCT-11-0248 21764905 PMC3170438

[16] Oliveira C, Sousa S, Pinheiro H, Karam R, Bordeira-Carrico R, Senz J, et al Quantification of epigenetic and genetic 2nd hits in CDH1 during hereditary diffuse gastric cancer syndrome progression. Gastroenterology. 2009;136(7):2137–48. Epub 2009/03/10. 10.1053/j.gastro.2009.02.065 . 19269290

[17] Sharma S, Lichtenstein A. Aberrant splicing of the E-cadherin transcript is a novel mechanism of gene silencing in chronic lymphocytic leukemia cells. Blood. 2009;114(19):4179–85. 10.1182/blood-2009-03-206482 19745069 PMC2774554

[18] Posthaus H, Dubois CM, Laprise MH, Grondin F, Suter MM, Muller E. Proprotein cleavage of E-cadherin by furin in baculovirus over-expression system: potential role of other convertases in mammalian cells. FEBS letters. 1998;438:306–10. 9827567 10.1016/s0014-5793(98)01330-1

[19] Karam R, Carvalho J, Bruno I, Graziadio C, Senz J, Huntsman D, et al The NMD mRNA surveillance pathway downregulates aberrant E-cadherin transcripts in gastric cancer cells and in CDH1 mutation carriers. Oncogene. 2008;27(30):4255–60. 10.1038/onc.2008.62 . 18427545

